# A framework and road map for rapid start-up and completion of a COVID-19 vaccine trial: A single clinical trial site experience

**DOI:** 10.1017/cts.2022.3

**Published:** 2022-01-12

**Authors:** Carlos Rojas, Stephen A. Spector, Bernadette Cale, Megan Loughran, Leander Lazaro, Eric Mah, Gary S. Firestein, Kathryn A. Gold, Mark Wallace

**Affiliations:** 1 Altman Clinical & Translational Research Institute, University of California San Diego, San Diego, California, USA; 2 Division of Infectious Diseases, Mother-Child-Adolescent HIV Program, University of California San Diego and Rady Children’s Hospital, San Diego, California, USA; 3 Mother-Child-Adolescent HIV Program, University of California San Diego, San Diego, California, USA; 4 Department of Medicine, Altman Clinical & Translational Research Institute, University of California San Diego, San Diego, California, USA; 5 Division of Hematology and Oncology, Altman Clinical & Translational Research Institute, University of California San Diego, San Diego, California, USA; 6 Division of Pain Medicine, Department of Anesthesiology, Altman Clinical & Translational Research Institute, University of California San Diego, San Diego, California, USA

**Keywords:** COVID-19 vaccine, study start up, clinical trial project management, rapid enrollment, scalable systems

## Abstract

The COVID-19 global pandemic required the rapid development of vaccines with a quick start up of phase 1–3 studies with large enrollment targets. The University of California San Diego was identified as a site for the phase 3 trial of the mRNA-1273-SARS-CoV-2 vaccine. There were many challenges with scaling up a large-scale clinical trial in such a short time. This report describes the processes and procedures that were implemented to successfully complete the enrollment target in under 10 weeks. This required the team to identify existing tools that could rapidly be accessed to develop a database, scheduling system, effective communication, document management, staff time tracking/efficiency, subject scheduling/tracking, project management, and accrual/study performance. The outcome of these efforts resulted in rapid enrollment and study completion in a short time. The lessons learned from this experience can be used by other clinical trial sites faced with similar challenges.

## Introduction

Coronavirus disease 2019 (COVID-19), caused by SARS-CoV-2, has spread around the globe causing more than 5,400,000 deaths [1] and devastating world economies. Shortly after the virus was characterized, multiple groups initiated programs to develop effective vaccines. mRNA technology was particularly promising, and two companies, Pfizer and Moderna, rapidly produced and tested their products. Initial phase 1 and 2 studies using the Moderna product, mRNA-1273, showed that the vaccine was tolerable and induced robust immune responses [2].

Plans for a large phase 3 registration study were quickly designed, intending to enroll 30,000 participants in a very short period of time. The University of California San Diego (UCSD) was identified as a site through the NIH-funded COVID-19 Prevention Network for the phase 3 trial of the mRNA-1273 SARS-CoV-2 vaccine (NCT 04470427). The UCSD Mother, Child and Adolescent HIV Program (MCAP) faced many challenges scaling up for the clinical trial with rapid and high volume accrual expectations, exacerbated by the urgency stemming from the COVID-19 global pandemic. Therefore, the Clinical and Translational Science Award-funded Altman Clinical and Translational Research Institute (ACTRI) partnered with MCAP to define the study resource needs, overcome hurdles, and devise a plan for high participant throughput. This report describes the processes and procedures that were implemented to enroll successfully 336 participants into the study in less than 10 weeks.

## Methods and Results

### Study Initiation

ACTRI and MCAP worked closely with the UCSD Office of IRB Administration to expedite the regulatory steps required for the mRNA 1273 phase 3 vaccine study. An initial challenge was to build a webpage to lay out the study details and make it available to the public. The website provided a link to a survey allowing potential volunteers to enter demographic data. Interest levels in the community were very high and the survey helped the team efficiently identify the most viable candidates.

REDCap©, a database developed by the University of Vanderbilt, was used for the initial interest survey and for key demographic data [3]. Over 5000 volunteers completed the intake form within 4 weeks, demonstrating outstanding local community interest. The ACTRI team narrowed the selection of participants based on protocol priorities (i.e., over 65 years old, ethnic diversity, health care workers). The data management team used REDCap analytical capabilities to flag priority characteristics from the volunteer database, then reviewed the resulting list with the Principal Investigator to proceed with outreach based on this rubric. This survey analysis identified a pool of potential participants to launch a screening effort.

Initially, participants were contacted by telephone. Coordinators called priority participants, answered their questions, and discussed consent forms. The process was time consuming and revisions to the methods were required to meet accrual goals so new tools were implemented to reach the target audience. Traditional Zoom© meetings, while widely accessible, were not appropriate for consent discussions because of the visibility of audience members and therefore potential exposure. Methods to reach many people at once, digitally, while exercising great control over visibility and interaction were required. Therefore, widely viewed Zoom webinars, which do not allow attendees to see one another to preserve privacy and HIPAA compliance, were used to inform a much broader audience in a short period of time.

**Fig. 1.**
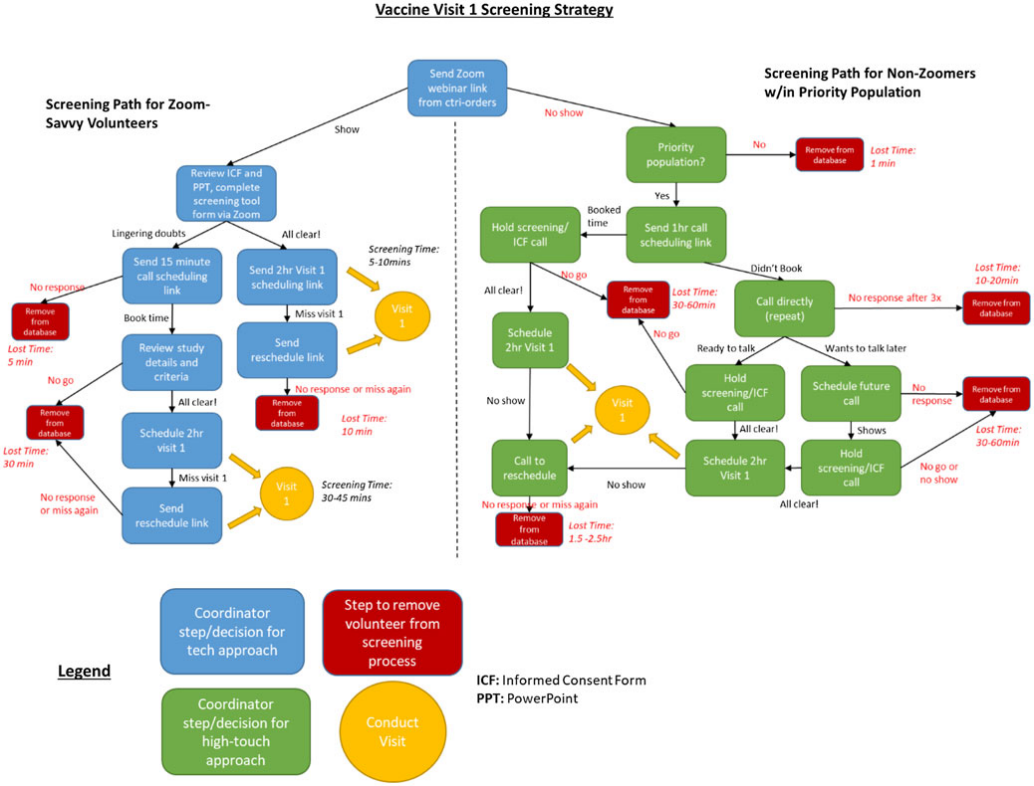
Two-pronged screening strategy to optimize coordinator time.

The Zoom webinars were effective for more internet fluent volunteers; however, we recognized that exclusive reliance on Zoom recruitment would miss potential participants from some high-risk groups. For less tech-savvy potential participants, additional coordinator and community representative time was reserved for better targeting. Thus, two different screening paths for volunteers were developed (Fig. 1).

### Scheduling

**Fig. 2.**
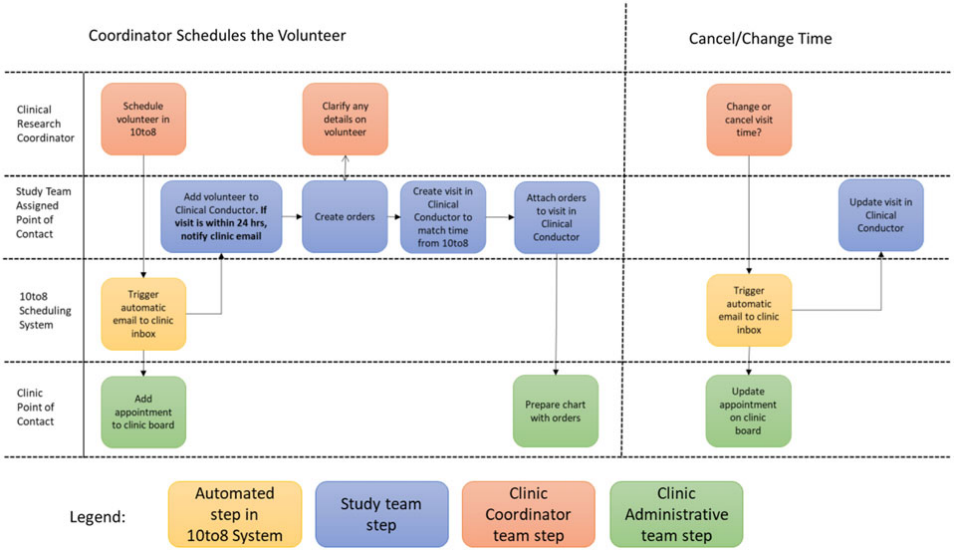
Process flow to aid teams with scheduling.

Scheduling large numbers of participants manually requires substantial coordinator time. This approach can be effective for small studies, but a lightweight tool was essential for enrolling hundreds of individuals (including recurring visits) over several weeks (Fig. 2). The team arranged demos, evaluated, and then selected 10to8.com©. The 10to8.com system was customized to accommodate the variety of visit types for this trial, consider staff and room requirements, and scheduling templates. This required adjustment of notification messages, frequency of updates, media (text, email, voice), and alerts on scheduling changes. Different colors were used to indicate different visit types, such as screening, randomization, visit 2, phone follow-up, and so forth. It was important to establish parameters for self-scheduling for advanced notice, so that subjects could neither change appointments within 24 h of a visit, nor reschedule themselves to a new time in under 24 h. This was important for resource management and drug stock level. An additional challenge was complying with visit windows after the open label phase. This created situations where subjects might have an appointment for an open label visit 1, open label visit 2, original visit 3, and original visit 4, each with different window requirements.

### Communication

Coordinating the many team members involved in the Moderna vaccine trial start-up required regular and seamless communication. The ACTRI team established weekly Zoom meetings to connect team members and resolve any pending issues. Questions that could not be addressed immediately were handled after the meeting. Microsoft Teams (MST) was selected as the most appropriate environment for daily real-time and asynchronous communication [4].

### Document Management

A OneDrive directory was used with permissions set at the folder level and was implemented for document management [5]. OneDrive allowed quick share links customized for access to only particular team members, or to broad swaths of the organization. This tool also had the advantage that it had an integration with the overall project management platform. Therefore, tasks could be created and managed in the project management tool while directly linking the task to document repositories for relevant materials in OneDrive. OneDrive provided updates to create automated notifications to increase visibility.

The MST channel provided daily real-time communication between administrative and patient-facing staff and allowed OneDrive documents to be automatically linked, creating more seamless collaboration and reducing the need to switch between applications. An example was updating staff certifications for CITI GCP. These were housed in OneDrive and could be shared with a click with other MST members.

### Staff Time Tracking and Efficiency

As part of overall data tracking effort on team utilization, Harvest.com© time tracking was used. Harvest.com is a flexible mobile app that can be deployed to team members and centrally managed. Team members tracked their time against the Moderna project and the data were reviewed and approved weekly. Harvest.com was used to charge coordinator, project management, and regulatory support time, as well as to identify which team members had capacity to take on additional tasks and to right size the research team. Fully scheduled days in the clinic required as many as 25 team members (coordinators, pharmacists, nurses, physicians, administrative staff) while half days during the vaccination phase still required at least 14.

### Scheduling and Visit Tracking

To estimate potential enrollment during the brief 9-week enrollment period, a spreadsheet linear optimization model was used. This model considered resources, rooms, and the requirements for each visit. It also sought to optimize the distribution between visit 1s and visit 2s since every subject enrolled would result in a visit 4 weeks later, leading to diminished capacity for new enrollment. This model gave a potential for 345 subjects over the enrollment period, of whom 336 were ultimately enrolled. It also accounted for cost differentials between overtime/Saturday and weekday afternoons, to provide guidance in blocking out scheduling.

The day-to-day flow was visualized on another spreadsheet, where each phase of the visit was portrayed, accounting for resources available for each step, along with the arrival flow of subjects. This helped plan for the number and type of practitioners to have available (Supplementary Figure 1).

### Project Management

Monday.com© assists with project management [6]. The initial use case was to create visibility on tasks, owners, due dates, and statuses. A Monday board was used that was reviewed in regular team calls to keep aware of needs. Automations were programed into tasks so that owners would be notified of coming due dates with anticipation. Other team members could receive notifications of completed tasks through these automations too. By switching the status on any task, the item moved through the workflow, to give a high-level view of items in progress, not started, stuck, or completed.

The Monday.com platform was then expanded to accommodate subject tracking throughout the study lifecycle. A Business Associate Agreement was signed with Monday.com to adhere to HIPAA requirements. With this completed, a highly interactive dashboard of subjects, payment status, visit dates, upcoming actions, notes, and other critical tracking details were built that coordinators and clinic staff could both access and edit. The platform provided full audit trail with time stamps to give central visibility into changes (Supplementary Figure 2).

Monday.com was also used to manage daily staffing challenges. The study required combinations of nurses, physicians, phlebotomists, pharmacists, coordinators, and support staff, based upon daily numbers of subjects and types of visits. As resources were drawn from multiple teams to meet the demand of this trial, a collaborative and flexible tool to work from was required. A Monday.com board was developed with the various roles and assigned team leaders to populate their daily staff member commitments (Supplementary Figure 3). This eventually provided a powerful dashboard of daily visit histories (Supplementary Figure 4).

### Accrual and Study Performance

**Fig. 3.**
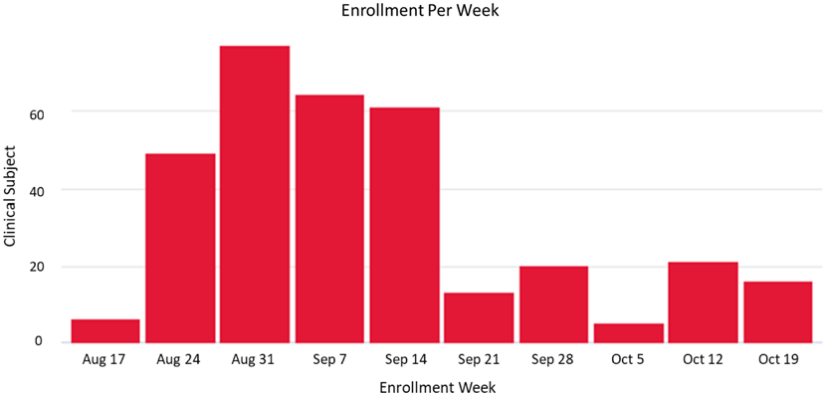
Actual enrollment for site by week; declining as participants return for second visits.

**Fig. 4.**
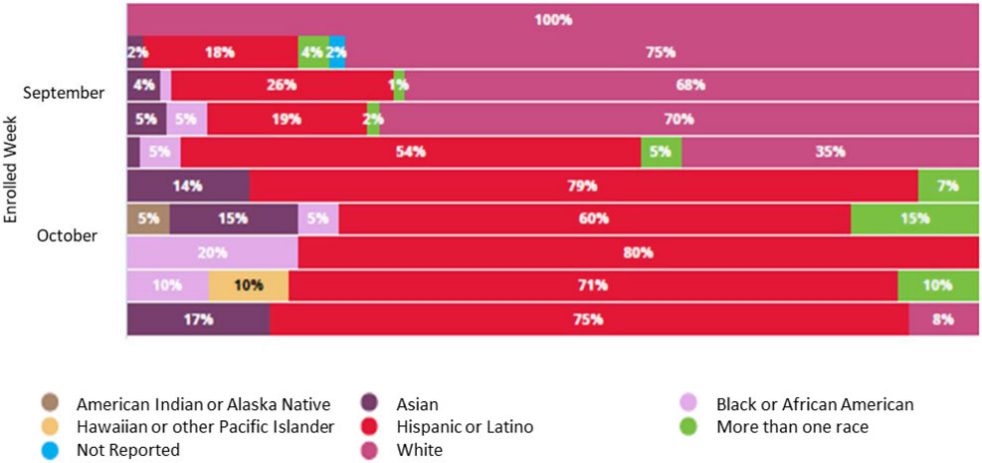
Actual demographics data for accruals by week.

The UCSD team accrued 336 subjects during the enrollment period of 65 days (Fig. 3). The study process initially focused on older adults but quickly pivoted to priority demographic groups (Fig. 4). By week 5 of accrual, the sponsor specifically requested that we emphasize underserved populations, leading us to shift our enrollment team, particularly Spanish-speaking coordinators, to prioritize our volunteer database to schedule accordingly. The data team sorted the volunteer database according to the new demographic requirements from the sponsor: underrepresented minority, specific zip codes, and under 65 years old. We halted scheduling any volunteers outside of this group to dedicate our resources to targeted outreach via phone and email. We created additional booking slots in the 10to8.com system to allow these priority volunteers to choose convenient times from a unique link sent to them. With continued use of flexible systems and spreadsheet modeling, visits were carefully grouped to minimize active vaccine wastage from vials. The study team had a list of last-minute backups to fill open spots after cancelations. Retention of volunteers was enabled through the 10to8.com HIPAA-compliant messaging system, with automations and notifications, along with dedicated follow-up from coordinator team members who gained capacity through the use of the various systems discussed. 93% of volunteers stayed on-study through the start of the open-label phase in January 2021. The successful completion across the country and results of this trial contributed to an emergency use authorization by the FDA for this vaccine in December 2020 [7].

As lessons learned throughout were documented, the study also provided guidance to partners at El Centro Regional Medical Center, as they looked to expand their clinical research operations and participate in vaccine and therapeutic trials. Our subject tracker templates were also provided to other studies working on the Janssen and AstraZeneca vaccine trials.

## Discussion

In December 2019, an outbreak of the COVID-19 emerged and over the next few months became a global pandemic affecting millions of people worldwide. The morbidity and mortality of COVID-19 led to international efforts to develop vaccines and therapies. These scientific collaborations resulted in the rapid deployment of candidate vaccines that were ready for human testing within months. Early phase results were promising, and vaccine candidates quickly moved into phase 3 testing [2].

During development of the phase 3 protocol, it became clear that we had to be prepared for rapid activation and unprecedented enrollment in this study. Here, we present our experience with this challenge and a framework that can be used to accomplish a rapid start-up and subject enrollment while at the same time maintaining high quality research. Extensive planning and coordinated efforts between the principal investigator, the investigator’s staff, and the research staff (including clinic managers, project managers, research clinic staff, and dedicated study coordinators) occurred even before a final protocol was available. By expediting regulatory processes and refining our accrual, consent, and scheduling processes, we were able to enroll 336 subjects to this study in 65 days. Communication between the team members was essential. A variety of software programs enabled collaboration and improved organization.

This process taught us both the importance of advance planning and also the need to remain flexible and modify processes that were found to be less effective or efficient than desired. For example, our team initially planned to reach out to potential subjects by telephone, but this was a time-consuming process. The process was changed to use virtual webinars to reach many participants, but allowed for flexibility to use a more personalized approach to reach out to certain target populations. Communication throughout the process was critical, and software including Microsoft Teams was used to ensure that relevant conversations were available to all involved team members.

Though this trial pulled staff support for other ongoing studies, we tried to minimize this negative effect by extending hours and utilizing afternoon, evening, and weekend hours. The COVID-19 pandemic has created unique challenges and opportunities in biomedical research. The rapid development of COVID vaccines is a success story and has saved many lives. The team’s outstanding performance implementing procedures and processes required for success can be leveraged by other institutions faced with similar challenges.

## Supplementary material

For supplementary material accompanying this paper visit https://doi.org/10.1017/cts.2022.3. click here to view supplementary material
