# Supplementary material for: A framework and road map for rapid start-up and completion of a COVID-19 vaccine trial: A single clinical trial site experience
Source: J Clin Transl Sci. 2022 Jan 12;6(1):e21. doi: 10.1017/cts.2022.3 (PMC8889226; doi:10.1017/cts.2022.3)
Supplement: Supplementary file 1 [file S2059866122000036sup001.docx]

**A Framework and Road Map for Rapid Start-up and Completion of a COVID-19 Vaccine Trial: A Single Clinical Trial Site Experience**

**Supplementary Material**


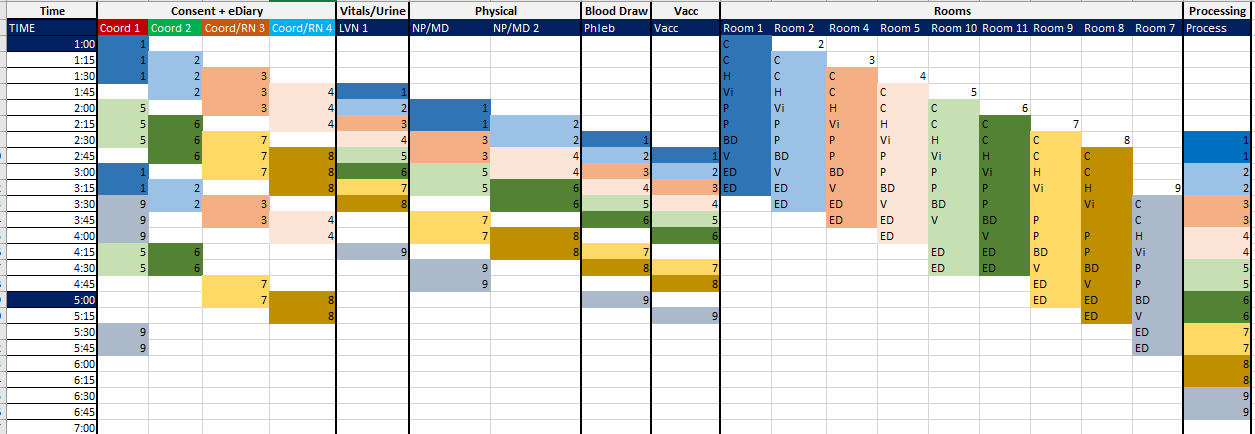

Supplementary Figure 1: Model for weekday flow of subjects, rooms, and resources


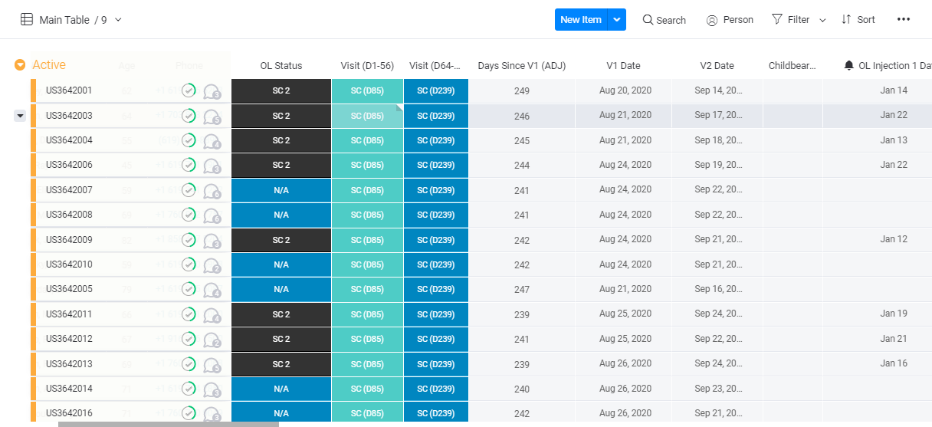


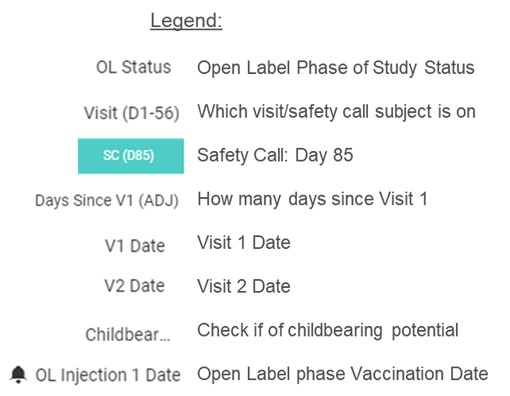


Supplementary Figure 2: Custom-built subject tracking tool on Monday.com


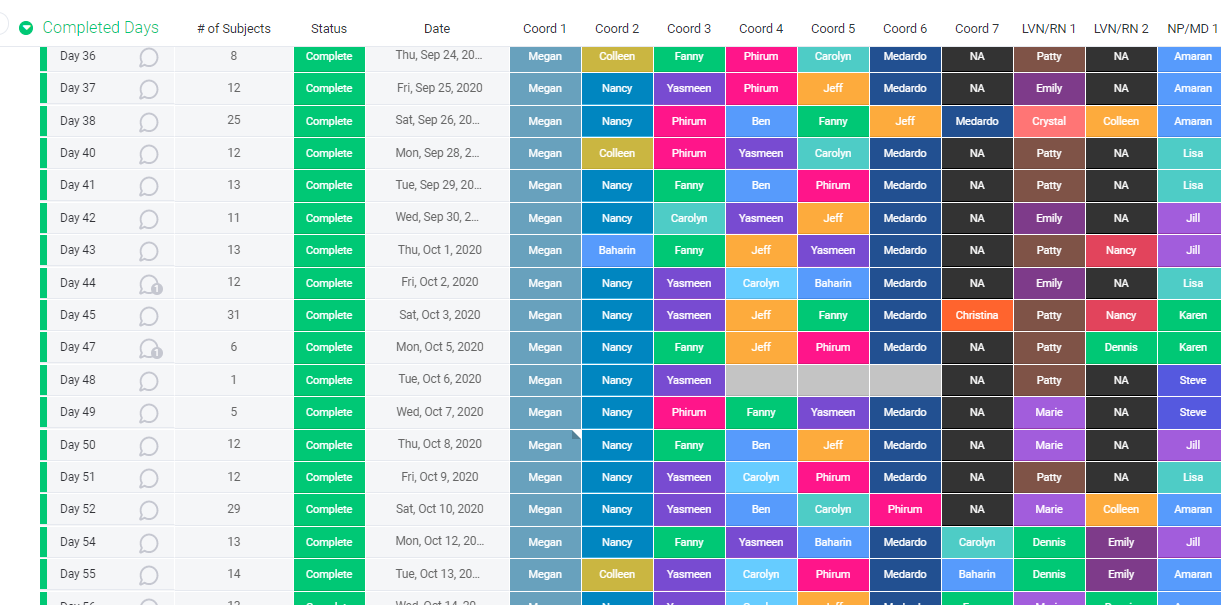


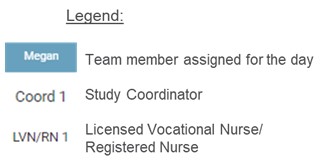


Supplementary Figure 3: Daily staffing calendar on Monday.com


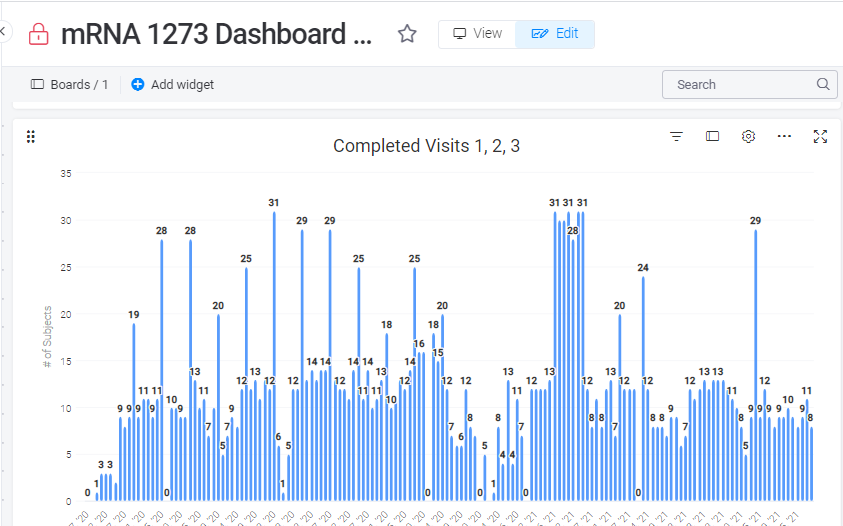


Supplementary Figure 4: Daily visit totals on Monday.com dashboard
